# A Death from Bromide of Ethyl

**Published:** 1895-08

**Authors:** 


### A Death from Bromide of Ethyl.

*L' Union Medicale*, May 8, 1894, reports that Mendoza reports a case of a death following anæsthesia produced by bromide of ethyl. Ten seconds after the application of the compress to the face the patient's color became cyanotic and the veins turgescent, the eyes were moved convulsively, the pupils were widely dilated. Rythmic tractions of the tongue and artificial respiration were practiced thirty minutes and active flagellations resorted to without effect. The cause of death seemed to be respiratory failure.

